# Improved non-destructive 2D and 3D X-ray imaging of leaf venation

**DOI:** 10.1186/s13007-018-0274-y

**Published:** 2018-01-19

**Authors:** Julio V. Schneider, Renate Rabenstein, Jens Wesenberg, Karsten Wesche, Georg Zizka, Jörg Habersetzer

**Affiliations:** 1 Department of Botany and Molecular Evolution, Senckenberg Research Institute and Natural History Museum Frankfurt, Senckenberganlage 25, 60325 Frankfurt, Germany; 2 Institute of Ecology, Evolution and Diversity, Goethe-University, Max-von-Laue-Str. 13, 60439 Frankfurt, Germany; 3 Department of Messel Research and Mammalogy, Senckenberg Research Institute and Natural History Museum Frankfurt, Senckenberganlage 25, 60325 Frankfurt, Germany; 4 Department of Botany, Senckenberg Museum of Natural History Görlitz, Am Museum 1, 02826 Görlitz, Germany; 5 German Centre for Integrative Biodiversity Research (iDiv) Halle-Jena-Leipzig, Deutscher Platz 5e, 04103 Leipzig, Germany; 6 International Institute Zittau, Technische Universität Dresden, Markt 23, 02763 Zittau, Germany

**Keywords:** Contact microradiography, Image processing, Leaf clearing, Micro CT, Vein networks, Vein density

## Abstract

**Background:**

Leaf venation traits are important for many research fields such as systematics and evolutionary biology, plant physiology, climate change, and paleoecology. In spite of an increasing demand for vein trait data, studies are often still data-limited because the development of methods that allow rapid generation of large sets of vein data has lagged behind. Recently, non-destructive X-ray technology has proven useful as an alternative to traditional slow and destructive chemical-based methods. Non-destructive techniques more readily allow the use of herbarium specimens, which provide an invaluable but underexploited resource of vein data and related environmental information. The utility of 2D X-ray technology and microfocus X-ray computed tomography, however, has been compromised by insufficient image resolution. Here, we advanced X-ray technology by increasing image resolution and throughput without the application of contrast agents.

**Results:**

For 2D contact microradiography, we developed a method which allowed us to achieve image resolutions of up to 7 µm, i.e. a 3.6-fold increase compared to the industrial standard (25 µm resolution). Vein tracing was further optimized with our image processing standards that were specifically adjusted for different types of leaf structure and the needs of higher imaging throughput. Based on a test dataset, in 91% of the samples the 7 µm approach led to a significant improvement in estimations of minor vein density compared to the industrial standard. Using microfocus X-ray computed tomography, very high-resolution images were obtained from a virtual 3D–2D transformation process, which was superior to that of 3D images.

**Conclusions:**

Our 2D X-ray method with a significantly improved resolution advances rapid non-destructive bulk scanning at a quality that in many cases is sufficient to determine key venation traits. Together with our high-resolution microfocus X-ray computed tomography method, both non-destructive approaches will help in vein trait data mining from museum collections, which provide an underexploited resource of historical and recent data on environmental and evolutionary change. In spite of the significant increase in effective image resolution, a combination of high-throughput and full visibility of the vein network (including the smallest veins and their connectivity) remains challenging, however.

**Electronic supplementary material:**

The online version of this article (10.1186/s13007-018-0274-y) contains supplementary material, which is available to authorized users.

## Background

Vein networks are one of the key structures of leaves and fulfil a series of important functions, including the transport of water and carbohydrates, providing mechanic stability, and resistance against herbivory [1]. They are also one of the key determinants in high photosynthetic capacity [2]. Without the network complexity achieved in angiosperms, photosynthetic capacity and evapotranspiration rates would not have reached the levels required to establish and maintain, for example, the highly productive megathermal tropical rainforests [3, 4].

The discovery of such linkages during the last years has led to increasing demands for leaf venation data to corroborate previous findings and to detect new relationships in a broad array of research fields ranging from plant physiology and systematics to macroecology and paleoecology. In spite of its importance to multiple research fields, the development of methods that allow rapid generation of large sets of leaf venation data has lagged behind, with the consequence that venation studies have been frequently data-limited [5, 6]; for available tools for image analysis, see [7, 8].

A major obstacle in research on leaf venation networks still is the dependence on chemical leaf clearing, a destructive, often multi-day method to visualize the network architecture [1, 6, 9]. Paradermal sections using a handheld razor blade with subsequent bleaching are an alternative, even though destructive method [10, 11], but are challenging for many species and only allow the removal of small areas of leaf tissue. Thus, none of these methods provide the required acceleration of the preparation process, nor the versatility to be applicable to leaves from herbarium specimens, which are an invaluable and still underexploited resource for leaf venation data and associated environmental information, but should not be subjected to invasive methods. To date, the most promising attempts have been made with X-ray technologies. X-ray technology is both fast and morphologically non-destructive (i.e. disregarding deleterious effects at the molecular level) and, thus, has the advantage of being also appropriate for leaf samples from herbarium collections.

Two basic issues have to be tackled with when using X-ray for imaging leaf venation networks. Firstly, because leaf tissue consists of low atomic number materials, photon absorption is generally low and decreases rapidly with increasing photon energy [12]. Low photon energies that correspond to the low Kα-resonance of leaf tissues, in turn, may not penetrate thick leaves [6]. Therefore, leaf texture and thickness will influence the X-ray based visualization of the vein networks. Moreover, the low contrast between veins and surrounding mesophyll tissue hampers the detection of veins (especially those with smaller diameters) and their connectivity. Low contrast also makes images less suitable for semiautomatic image analysis (5). Secondly, the resolution of the X-ray detector [6] or the image plate (this study) combined with the properties of the X-ray system (e.g. geometric magnification, spot size) sets a limit to the maximum resolution which may lie in or above the range of the size of the smallest veins [6, 13]. To increase the contrast of plant tissues, contrast agents such as iodine may be applied to samples [6, 14, 15]. However, the disadvantage of applying contrast agents are an increase in preparation time, potential creation of artefacts and degradation of samples [13, 15], which should be avoided in herbarium specimens.

Wing [16] was the first to test analogous 2-dimensional (2D) X-ray imaging of leaf venation networks and obtained well-resolved vein networks in about half of the samples with multi-minute exposure times. The utility of modern medical diagnostic 2D X-ray instruments was assessed more recently, but there was no significant improvement because of persistent limitations imposed by their resolution [6]. This highlights the need for improving the detector’s resolution beyond the industrial standard of 25 µm to visualize also the smaller veins while keeping the benefits of higher throughput imaging provided by rapid 2D X-ray systems.

Synchrotron X-ray imaging has recently been successfully used for high-throughput production of high-resolution vein images [6, 17]. However, synchrotron light sources are not readily accessible and are therefore no option for routine imaging. Alternatively, high-resolution images superior to those of 2D X-ray instruments can be produced with 3-dimensional (3D) computed tomography (micro CT) as shown in an initial attempt [6]. To date, a comprehensive testing of micro CT for leaf vein imaging is still lacking, including a quantitative comparison with 2D X-ray imaging at different resolutions, the avoidance of a contrast agent and the development of an image processing standard, which would greatly improve the repeatability of imaging experiments and allow for more widespread use of micro CT systems in leaf venation studies.

## Materials and methods

Although there has been substantial progress in our understanding of the applicability of modern X-ray systems for the study of leaf venation, several issues and technological approaches have not been addressed to date. In this study, we aim at (1) developing and testing new approaches in 2D X-ray scanning technology (i.e., increasing image resolution beyond the industrial standard of 25 µm by using thinner image plates) for non-destructive high-throughput routine visualization of vein networks from herbarium samples and (2) improving 3D X-ray methodology for the production of very high-resolution images of vein networks without contrast agents (including an acceleration of scanning time, i.e. parallelization of imaging) that will be suitable for a variety of applications and research questions. Moreover, we develop imaging standards comparing the suitability of approaches across a broad array of leaf and leaf venation types in vascular plants for quantitative measurements of vein traits.

## Taxa, leaf samples, and morphological descriptors

Leaf samples were taken from herbarium specimens of taxa that differ with respect to leaf texture, pubescence, thickness and venation types in angiosperms. All samples are from the herbaria FR, GLM, L, LZ, U, and WAG (abbreviations according to [18]). For quantitative comparisons of the different vein imaging methods, we used a set of 33 species (Table 1). Only well-developed flat leaves were chosen. Entire leaves as well as fragments of approximately 1 cm diameter were used as samples. The leaf fragments included a primary or secondary vein as reference point for subsequent classification of vein orders and, in larger leaves, were taken from about the middle of the leaf between the midvein and the leaf margin.

**Table 1:** Voucher information and differences in the resolution of leaf veins as assessed from vein density (VLA) calculations based on two different 2D X-ray image plate resolutions, the industrial standard (25 µm) compared with our new project standard (7 µm) as well as micro CT (CT)

Taxon	Collector and collection no. (herbarium acronym)	2D X-ray: 25 µm versus 7 µm		CT versus 2D X-ray (7 µm)		CT image with full vein resolution
		Mean VLA (mm mm^−2^) 25/7 µm	% similarity in VLA calculations	Mean VLA (mm mm^−2^) 7 µm/CT	% similarity in VLA calculations	
*Anemia abbottii* Maxon	Bisse 27767 (FR)	1.98/1.98	100.0	1.98/1.98	100.0	Yes
*Bobgunnia madagascariensis* (Desv.) J.H. Kirkbr. and Wiersema	Höhn 19 (FR)	5.26/6.85	76.8***	6.85/11.5	59.6***	Yes
*Boscia senegalensis* Lam.	Kahlheber 497 (FR)	3.99/4.43	90.1	4.43/4.93	89.9	Yes^b^
*Burkea africana* Hook.	Zwarg 81 (FR)	6.35/7.69	82.6***	7.07/8.16	86.7***	Yes
*Daniellia oliveri* (Rolfe) Hutch. and Dalziel	Kéré 822 (FR)	2.88/3.41	84.5*	3.41/8.31	41.1***	Yes^b^
*Detarium microcarpum* Guill. and Perr.	Küppers 636 (FR)	4.02/5.20	77.3***	5.20/6.66	78.1***	Yes
*Euthemis leucocarpa* Jack	Fuchs 21192 (L)	1.64/1.68	97.6	1.68/7.27	23.1***	Yes
*Froesia venezuelensis* Steyerm. and G.S. Bunting	Schneider 1 (FR)	n.d.^a^	n.d.^a^	n.d.^a^	n.d.^a^	Yes
*Idertia axillaris* (Oliv.) Farron	Bissiengou 1275 (WAG)	3.48/4.20	82.8*	4.21/8.79	47.9***	Yes
*Lophira lanceolata* Tiegh. ex Keay	Neumann 1530 (FR)	2.41/2.75	87.6*	2.75/4.76	57.8***	Yes^b^
*Mirabilis prostrata* (Ruiz and Pav.) Heimerl	Schneider 2842 (FR)	0.87/0.87	100.0	0.87/1.35	64.4***	No^c^
*Ochna afzelii* R. Br. ex Oliv.	Neumann 923 (FR)	3.35/5.48	61.2***	5.58/13.28	41.24***	Yes
*Quercus faginea* Lam.	Benavides SP13_QUFA46	3.53/4.67	75.4***	4.67/8.06	58.0***	Yes
*Rhabdophyllum calophyllum* (De Wild. and T. Durand) Tiegh.	Hijmans 424 (L)	2.95/5.36	55.1***	5.36/7.62	70.3***	Yes
*Acer pseudoplatanus* L.	Benavides RO025	3.96/4.38	90.5	na	na	na
*Afzelia africana* Sm.	Küppers 1951 (FR)	2.86/3.30	86.9*	na	na	na
*Bauhinia rufescens* Lam.	Kahlheber 262 (FR)	4.26/5.68	75.0***	na	na	na
*Betula pendula* Roth	Benavides FIN03	3.54/4.45	79.5**	na	na	na
*Brackenridgea zanguebarica* Oliv.	Schultka 147 (FR)	3.11/3.65	85.2	na	na	na
*Bridelia ferruginea* Benth.	Hahn 978 (FR)	4.72/7.41	63.7***	na	na	na
*Cassia sieberiana* DC.	Kahlheber 925 (FR)	1.65/2.38	69.2***	na	na	na
*Cassia singuena* Delile	Hahn-Hadjali 1206 (FR)	2.92/3.91	74.8***	na	na	na
*Cristaria integerrima* Phil.	Schneider 3027 (FR)	4.61/7.37	62.5***	na	na	na
*Crossopteryx febrifuga* (Afzel. ex G. Don) Benth.	Küppers 796 (FR)	4.48/5.36	83.7***	na	na	na
*Diospyros mespiliformis* Hochst. ex A. DC.	Küppers 2131 (FR)	3.49/4.41	79.2***	na	na	na
*Godoya antioquiensis* Planch.	Uribe 2700 (U)	8.51/10.72^d^	79.4***	na	na	na
*Landolphia heudelotii* A. DC.	Neumann 738 (FR)	4.35/4.55	95.5	na	na	na
*Lophira alata* Banks ex C.F. Gaertn.	Leonard 1089 (FR)	1.98/2.71	73.3*	na	na	na
*Medusagyne oppositifolia* Baker	Friedmann 4444b (FR)	5.09/5.54^d^	91.8**	na	na	na
*Ocotea calophylla* Mez	Schneider 1238 (FR)	5.16/5.61	92.0**	na	na	na
*Ouratea scottii* Sastre	Jansen-Jacobs 1895 (U)	4.21/6.25	67.3***	na	na	na
*Ouratea vaccinioides* Engl.	Seele 736 (LZ)	2.29/3.17	72.4***	na	na	na
*Saba senegalensis* (A. DC.) Pichon	Schmidt 892 (FR)	3.03/3.57	85.1*	na	na	na

*Differences significant at *p* < 0.05/2, ** 0.01/2 or *** 0.001/2 level (after Bonferroni correction)

^a^The structure of the very dense, parallel last order veins was not high enough in 2D X-ray images for vein density measurement

^b^Some last order veins with low contrast

^c^Highest order veins not distinct

^d^The 7 µm images provide full resolution of the leaf venation (as identified by comparison with cleared leaf images)

## X-ray imaging of leaf venation networks

### 2D X-ray imaging

Leaf venation networks of entire leaves were visualized using high-resolution X-ray technology. Well-preserved, ideally flat leaves were placed on flexible image plates with a resolution of 25 µm, which is the industrial standard (as certified by the German Federal Institute for Materials Research and Testing, BAM, Berlin), and scanned in a Faxitron X-ray system (Hewlett-Packard) with a field of view of maximally 300 mm in diameter. Depending on the individual leaf size, up to 10 (typically 6-8) leaves were placed on an image plate. To achieve high-quality images, only leaves that provide direct contact with the image plate (the distance between both must not surmount 4–5 mm) were selected. In a first step, X-ray conditions were optimized using a test series with varying exposure times and photon energies. Because leaves contain elements with low atomic numbers, best contrast is achieved at low photon energies. Therefore, test images were generated with tube voltages between 12 (i.e. the lowest feasible tube voltage) and 30 kV and exposure times between 30 and 1020 s. Highest contrast was generally obtained with tube voltages close to the lowest limit and 600–1020 s. Image data were read with a semi-automatic image plate scanner (Dürr, Germany) and stored as 16-bit RAW and 8-bit BMP files (the latter for image size definition of the RAW import). To minimize deleterious effects from daylight during sample removal, X-ray and scanning was conducted under very low ambient light in a dark room with unshielded image plates.

In addition to the industrial standard of 25 µm resolution, we developed a new approach that led to a more than three-fold increase in resolution (hitherto called project standard). This was achieved by using a thinner image plate (UHD, ultra high definition with 20 µm nominal resolution) and hard- and software adaptations of the X-ray laser scanner system respectively (with the latter, we achieved a resolution of 7 µm). The hardware adaptations comprised an exchange of the normal image plate feeder with a special feeder in the scanner system (provided by Dürr, Germany) that allowed us using these UHD plates without failure of the image plate transport mechanism of the scanner or at least loss of scanning lines due to transport problems. The image intensifier and the laser beam intensity were set to achieve a nominal resolution of 20 µm. The highest resolution with a pixel size of 7 µm was achieved by additional software adaptations of the image acquiring modus of the X-ray laser scanner (i.e. a stepwise reduction of scan-speed which results in a higher spatial resolution) together with a low setting of the laser beam intensity combined with an extremely low tube voltage of 12 kV (resulting in a “superficial” scan of the image plate´s scintillator layer, which has the same effect as a further reduction of the scintillator´s thickness).

### 3D X-ray imaging

In the X-ray cabinet of the phoenix nanotom CT (General Electric), the Focus-Detector-Distance (FDD) was set to 200 mm only in order to obtain a strong low noise signal on the detector. The sample holder was placed directly in front of the microfocus X-ray tube and thus the geometric enlargement of the sample was up to eight-fold on the detector (pixel size 50 µm) resulting in an effective resolution of about 6.25 µm.

**Fig. 1:**
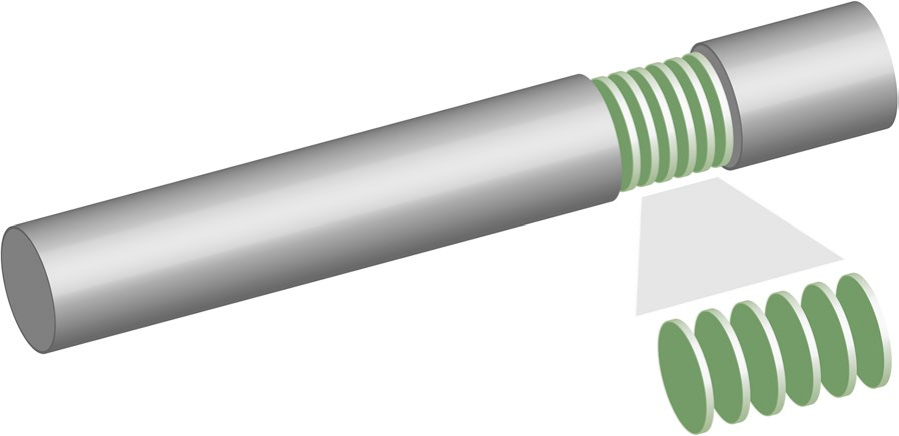
Tubular sample device used for imaging multiple leaf samples (here, circular sections) in a single micro CT scan

To increase the sample throughput of micro CT, we constructed a tubular sample holder made of paper and inserted up to 25 leaf samples. We tested each sample separated from the next by a polyethylene or polyurethane spacer (Fig. 1), specimens simply in direct contact with their neighbours, and samples arranged in groups of five specimens, with each group separated by a spacer for easier counting the leaf stack. The latter procedure turned out to be the most suitable for the consecutive separation of individual samples during the segmentation process.

After 360° rotation with 2400 projection images, the specimen holder was vertically lifted by half of the field of view for the next complete rotation, the latter of which resulted in overlapping 3D volumes at the end of the volume reconstruction procedure. It turned out that samples that were close to the centre beam (i.e. close to the centre of the field of view) had a superior CT scan quality compared to reconstructions from peripheral areas of the volumes. Here the oblique X-ray beam caused fading of very low density parts of the samples (Additional file 2: Fig. S2). Thus, a “mass” production of CT scanning becomes limited by quality demands, but still up to 15 samples could be processed simultaneously with sufficient image quality.

A great advantage of the CT method is that a single projection image may contain only very few grey scale steps (e.g. tube voltages 40–80 kV or even higher can be used) but then 2400 images were superimposed during the reconstruction process and so the number of grey values considerably increased. Moreover, with higher voltages, measurements become faster. In contrast, 2D radiography has to be performed with extreme soft radiation below 20 kV (see above). Virtual 2D processing of the CT raw data (based on a 3D image stack of multiple slices with a slice thickness of the chosen resolution, i.e. pixel/voxel-size of 6.25–25.0 µm) produces a kind of (2D-)radiography and was performed with the 3D-CT software VGStudio MAX, versions 2.6 and 3.0.1 (Volume Graphics GmbH, Heidelberg). Two program algorithms were used (“Sum along Ray” and “Maximum Projection”) and the resulting grey values were inversed to make the background white. (Inversed) “Sum along Ray” casts one ray per final (2D-)pixel into the data set. In other words, the density values are collected along a line of vision (ray) through the selected sub-volume. The lower the integrated density values of the voxels along this ray line through the slice image stack, the brighter the corresponding final pixel. The (Inversed) “Maximum Projection” algorithm also casts one ray per final pixel into the image stack. However, only the maximum intensity (i.e. the leaf tissue with the highest density) of the voxels along a ray determines the low grey value of the corresponding pixel. Although clearly differing in their algorithms, both procedures resulted in very similar high quality (low noise) imaging of the vein architecture with a very high contrast to the parenchymatic mesophyll. However, both approaches rely on initial time-consuming 3D rendering of data.

### Image processing standards and storage in public databases

A standard for subsequent image processing was developed with Adobe Photoshop, version CS6 13.0.1 × 64, with the aim of enhancing the contrast of the venation against the remaining leaf tissue and the background, making use of the wide greyscale range of the 16-bit images, and maximizing the uniformity of the images to facilitate subsequent manual or automatic vein analysis and for the storage of the leaves in public databases as well.

**Fig. 2:**
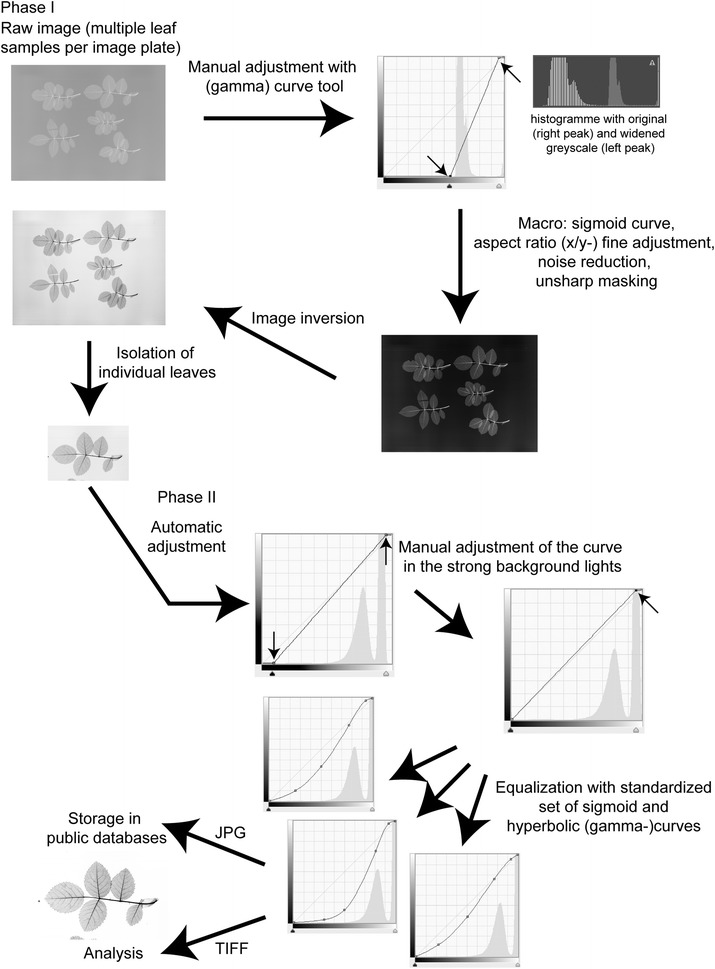
Image processing workflow for 2D X-ray images of leaf venation

Image processing was subdivided into two phases, each comprising 4 steps (Fig. 2) as outlined below. In the first phase (Phase I), images were manually adjusted (step 1) using the (gamma) curve tool. This adjustment consisted in shifting the black (shadows) and white areas (highlights), thereby eliminating for example approximately the uppermost ten percent of the tonal range, which are not represented in the images (these are confined to the image plate margins, e.g. X-rays of fixation tools). The result is a spreading of the tonal range of the leaf venation generating a higher contrast. It also serves as the preparation of the automatic application of a macro (step 2). This subsequent automatic adjustments with a macro includes 4 substeps (sigmoid curve, aspect ratio (x/y-) fine adjustment, noise reduction, unsharp masking filter) followed by conversion of the negative into a positive image (step 3) and isolating single leaf images from the multi-leaf arrangement of the raw images (step 4).

In the second phase (Phase II), the individual leaf images were subject to an automatic adjustment of the curve (here especially histogram details in the shadows which are visually not detectable; step 5). Very rarely, this step led to an overexposure of the non-veinous leaf tissue and was thus omitted. Next is a manual adjustment of the curve in the strong background lights (step 6). Finally, after the last equalization process with a standardized set of sigmoid and hyperbolic (gamma-)curves (step 7) the images were stored as TIFF and JPG images (step 8), the latter for upload in public databases. In summary, only two manual settings are needed whereas most of the image processing was done with a standardized macro and preselectable standardized gamma curves.

Image files have been made accessible through the Cleared Leaf Image Database [19] and partially in the Senckenberg collection management system Aquila [20].

## Chemical leaf clearing

For the comparison with traditional chemical methods of vein imaging, the leaf fragments subjected to X-ray imaging were kept and subsequently used for chemical clearing following [21]. The samples were placed in a biopsy cassette with a chamber size of 31 × 26 mm (Swingsette, Carl-Roth, Germany). These cassettes were transferred into a glass jar with an aqueous solution of 5% NaOH and heated (80 °C) for 2 h to 7 days until leaf fragments became transparent and soft. Afterwards, the samples were washed in distilled water and placed for 5–15 min in an aqueous bleaching solution of 2.5% sodium hypochlorite until the leaf had turned pale white. The samples were washed again and transferred (within the cassettes) into 50% ethanol. After 30 min, samples were brought into a staining solution (1% safranin O in ethanol, w/vol) for about 5–15 min. Then, samples were destained in 99% ethanol for at least 30 min and mounted on a glass slide in Roti-Mount (Carl Roth, Germany). Images were taken at 25 × and 63 × magnification using a Dialux 22 light microscope (Leitz, Wetzlar, Germany) and the Leica DC300 camera system. Calibration was done with the measurement module of the IM1000 software, version 1.20, release 9 (Leica Microsystems, Heerbrugg, Switzerland).

## Vein measurements, evaluation criteria and statistics

A gold mask with standardized line pairs and edged dot patterns was used as a control of the quality of the X-ray imaging methods (for example, to verify that resolution was 7 µm in our high-resolution 2D X-ray approach). Because nominal resolution of each of the X-ray methods is not necessarily the same as their resolution of the leaf venation (due to the properties of the biological tissue such as the impact of leaf texture; see Background section), especially of the finest veins, we additionally assessed differences between methods using a quantitative approach. We selected vein density as a measure that depends on the quality of the vein display. We calculated the vein density in 5–10 rectangles with identical position and size using the same leaf fragment for each taxon (for example, we compared 2D X-ray imaging with 25 µm resolution with that of 7 µm; Additional file 1: Fig. S1). To obtain vein density values, the length of all veins in each of the areas was traced digitally and measured using ImageJ [22] and the plugin ObjectJ, version 1.03x [23]. Digital tracing is still the method of choice and proved more accurate than semiautomatic measurements with LeafGUI [5, 24]. Significance of differences in vein density measurements between the different imaging methods was assessed with a paired *t* test in R [25]. A quantitative comparison of micro CT images with those obtained from chemically cleared leaves was hampered by the slight sample distortion during preparation that led to size differences of the objects. Therefore, we opted primarily for a visual comparison, verifying vein connectivity and the presence of vein orders.

## Results

### 2D X-ray imaging

For special non-destructive testing (NDT) tasks in industrial applications, an image plate of 20 µm resolution can be used by adapting the X-ray laser scanner settings to the corresponding x/y-resolution (i.e. increase of the pixel clock for the x- and decrease of the scan speed for the y-axis). Interestingly, we did not observe the expected loss of sensitivity for the newly adapted system, although the X-ray storage layer of the image plate is thinner and thus less sensitive. In addition, the area for capturing the X-ray photons is smaller. Obviously, the very soft radiation penetrated only the upper microns of the storage layer and so the thickness plays a minor role. On the other hand, the decrease of the scanning speed led to a higher output of laser induced (blue) light photons by more and longer laser excitations of the image plate. This observation encouraged us to further increase the resolution up to the micro-mechanical limits of the laser scanner system, which proved to be at 7 µm pixel size. Using testing tools (gold mask with standardized line pairs and edged dot patterns) we found this increase of resolution physically effective as well as the visibility of minor veins clearly increased. Thus, besides a resolution of 20 µm (plate size limit 18 × 24 cm) another high-end standard of 7 µm (plate size limit 10 × 12 cm) was established.

For the quantitative assessment of our X-ray methods, we compared measurements of VLA from vein images obtained with the industrial standard (image plate resolution of 25 µm) with those of our high-end project standard (7 µm resolution). With the 25 µm images fewer veins were traced unambiguously, resulting in lower values of VLA compared to the 7 µm images. These differences translate into a proportion of 55–100% of the vein densities recovered with 25 µm imaging compared to our new standard (Table 1). Only in seven samples these differences were not significant according to the paired t-tests. Three vein images with 7 µm resolution permitted the tracing of all veins unambiguously (Table 1), the rest revealed some degree of uncertainty in recognizing the last order veins, although the network architecture at large was distinct.

**Fig. 3:**
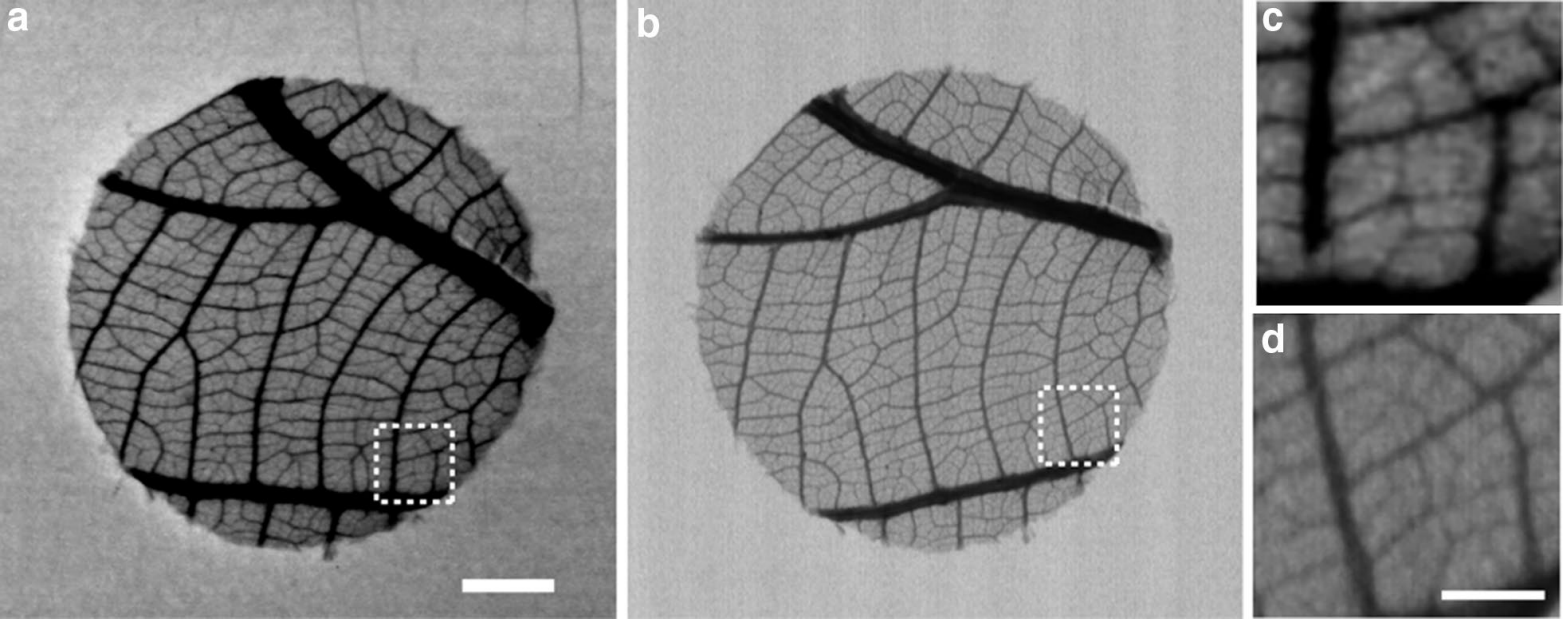
2D contact microradiography images of the leaf venation of *Bridelia ferruginea*: a comparison of the effect of 25 µm versus 7 µm resolution. **a** leaf venation at 25 µm resolution (rectangle indicates enlarged section shown in C). **b** leaf venation at 7 µm resolution (rectangle indicates enlarged section shown in **d**). Scale bars 5 mm (**a**, **b**) or 0.5 mm (**c**, **d**)

Apart from the differences in vein densities, another major difference between both image resolutions was the display of vein thickness. In the 25 µm images, vein diameters were artificially enlarged as evident from a comparison with the 7 µm images (Fig. 3) and cleared leaf images.

### Micro CT and a comparison with 2D X-ray images

**Fig. 4:**
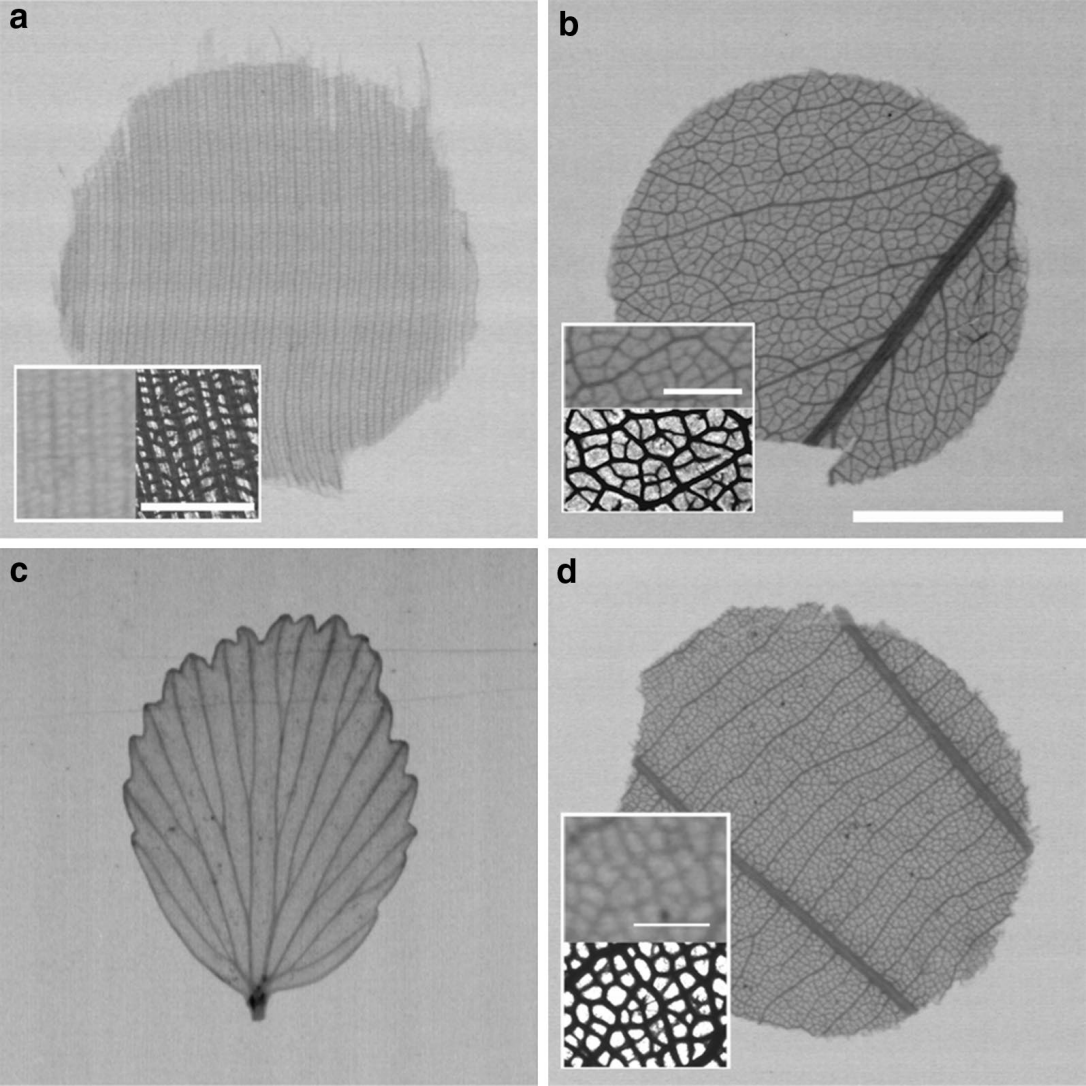
2D contact microradiography images (7 µm resolution) of selected leaf venation samples with full resolution of the vein networks. **a** *Elvasia calophyllea*: inset panel with details. **b** *Medusagyne oppositifolia*: inset panel with details. **c** *Anemia abbottii*. **d** *Godoya antioquiensis*: inset boxes show enlarged details together with identical or similar leaf sections of cleared leaf images (bottom or right side) for the assessment of maximum vein resolution. All images at same scale (scale bar 5 mm); scale bar of inset images, 1 mm

In a subsequent step, we imaged all samples using micro CT at a nominal resolution of 6.25 µm. Vein density measurements were done in the same way as for 2D X-ray imaging but only for a subset of the taxa. Here, at a per sample comparison, our 7 µm approach produced images that allowed 23.1–100.0% of the VLA to be traced unambiguously, compared with micro CT. The lower VLA values from 7 µm images arise from the incomplete display of the highest vein orders, particularly the free-ending veins (FEV), if present. Nevertheless, the full structure of the venation was visible in several species of our taxon set using 2D 7 µm images (e.g., *Anemia abbottii*, *Medusagyne oppositifolia*; Fig. 4).

**Fig. 5:**
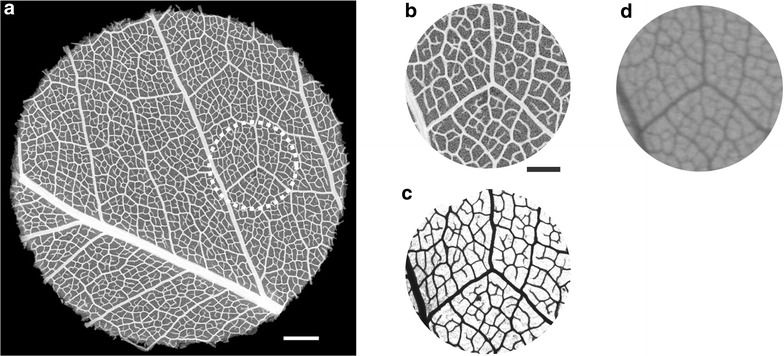
Very high-resolution imaging (virtual 2D) of leaf venation using micro CT. **a** Micro CT image of a leaf fragment of *Burkea africana* (Fabaceae); the circle refers to the enlarged view in **b**. **b** Detail of leaf venation with full resolution of minor veins. **c** leaf venation image from chemical leaf clearing; here, the same area as in **b** is shown. **d** image from 2D X-ray with 7 µm resolution. Scale 1 mm (**a**) or 0.5 mm (**b**–**d**)

**Fig. 6:**
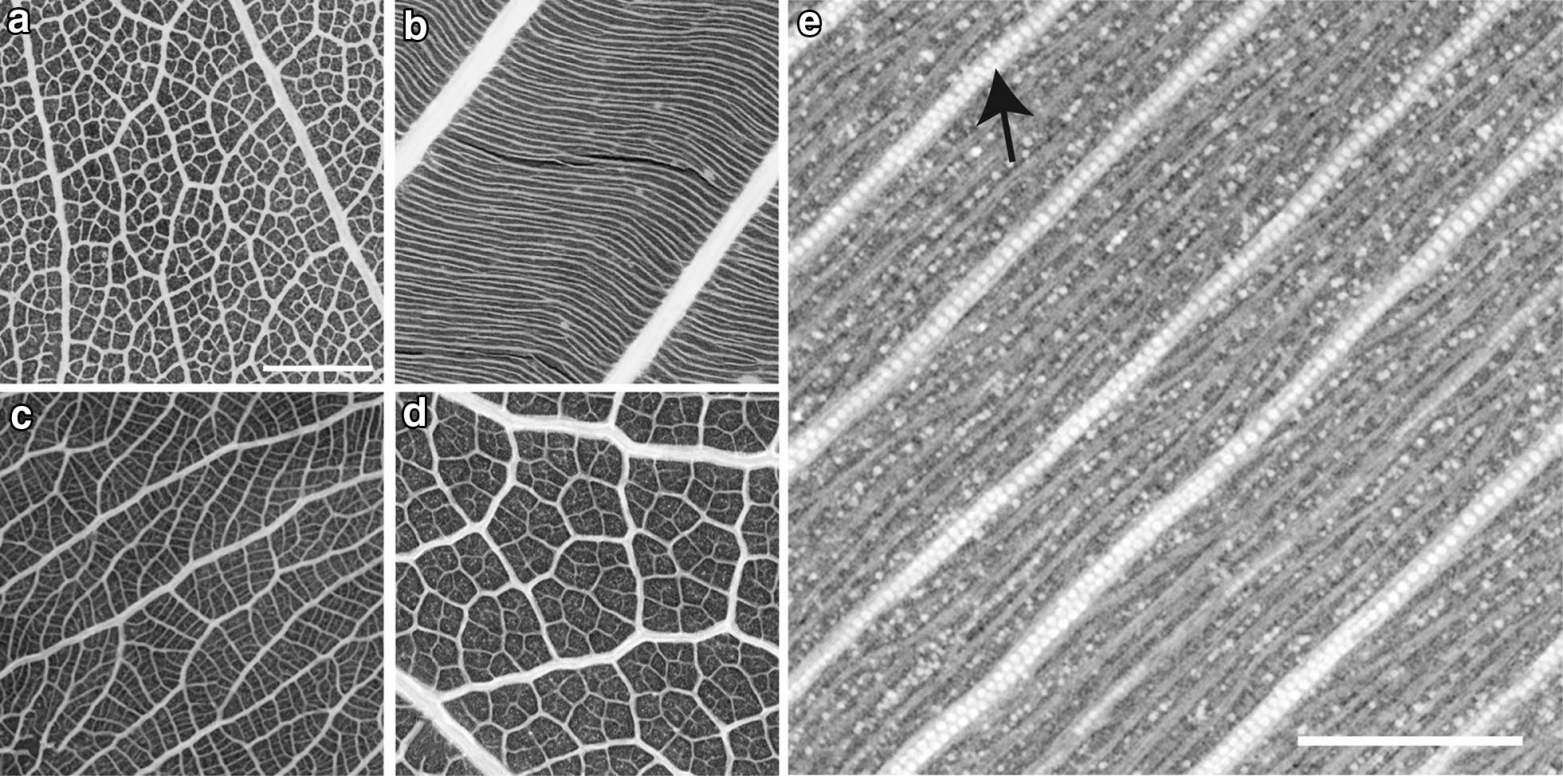
Enlarged sections of very high-resolution imaging (virtual 2D) of fully resolved leaf venation using micro CT. **a** *Bobgunnia madagascariensis*. **b** *Froesia venezuelensis*. **c** *Ochna afzelii*. **d** *Quercus faginea*. **e** *Euthemis leucocarpa* with crystals along secondary veins (arrow; light structures). Scale bar 1 mm (**a**–**d** at same scale)

Comparing micro CT with cleared leaf images revealed that the majority of micro CT images provided full resolution of the leaf venation (Table 1, Figs. 5, 6). Some of the micro CT images even provided more distinct vein patterns than in cleared leaves because of more homogenous vein contrast. In some micro CT images, however, the FEVs were partially difficult to discern unambiguously (e.g. *Daniellia oliveri*; Table 1).

### Image processing standards and contrast increase

Initially, a simple image processing approach (“quick approach”) was tested: Here, image processing comprised three steps only. In the first step, a digital master image was produced by converting the RAW file in TIFF format with a resolution of 1007 dpi and inverted grey scale. In the second step, an improved working image was produced by gradation adaptation of the range of grey scale values setting a sigmoid curve (without influencing leaf venation details) and isolating the object. In the last step, image sharpness was increased by applying a filter that is adapted to the main characteristics of the image plates (mask intensity = 150–200%, radius = 2.5 pixel, threshold = 0). Disturbing image artefacts were minimized by choosing settings of the mask that are below the optimal settings for the display of object details. The drawback of this procedure was a specific increase of the width in fine texture lines due to non-linear (local) contrast enhancement which is sufficient for the visual detection but less suited for measurements. This is the reason why in the two-phase (multi-step) procedure used subsequently (see Methods), the sharpness filter is only used for the compensation of blurred image details by the Gaussian noise filter with a quite weak mask intensity (= 50%) and a very small radius (= 0.2 pixel, threshold = 0).

The here established two-phase processing standard ultimately proved most suitable because it allowed for adjustments according to leaf texture while keeping the processing time in a range that is appropriate for higher throughput. The distribution of grey tones in the X-ray images of the vein networks showed two mostly non-overlapping peaks, the first representing the leaf tissue, the second the background. Thus, the curve could be shifted to cut off part of the right (i.e. the lighter) margin without losing information. To this point, all adjustments consisted in a manipulation of the linear curve, thereby maintaining equal distances between adjacent grey tones. Subsequent enhancements were done with a sigmoidal curve that allowed us to decrease the contrast of the grey tones of the background (i.e. flattening the curve for the lightest part of the greyscale), which are not informative. By increasing the slope of the curve for the grey tones of the leaf tissue, the contrast of the venation was further enhanced without creating tonal breaks. To cope with varying degrees of vein contrast arising from differences in leaf structure (thickness, texture, etc.) and the degree of lignification of the veins, we designed three different sigmoid curves for our standardization of image processing (as summarized above in step 7). For thinner leaves with poorly contrasting veins, the slope of the curve covering the object was increased, whereas the curve was more flattened at the lightest part that corresponds to the background. For leaves with greater contrast, i.e. more distinct veins, the slope of the curve covering the object was rendered less steep (Fig. 2). For this fine-tuning step, it is important to avoid tonal breaks that may lead to the loss of vein connectivity and thus impair the utility for vein analysis. Some thin leaves showed very low contrast against the background (with a single peak in the histogram). In these cases, a manual increase of the tonal range is not recommended because of overexposure of the non-veinous leaf tissue. Instead of a sigmoidal curve a rather exponential curve was applied to avoid a flattening in the lightest part of the tonal range which would cause the loss of object information (see step 7 described under Methods).

Besides this general procedure of standardization some specific characteristics were subject to further tests: Because of the slightly asymmetric conical X-ray beam the exposure across the image was uneven, with the lightest part being in the centre. An additional effect of imbalanced exposure may arise from minimal light impact during sample removal, rendering the proximal image plate margin lighter compared to the opposite margin that is more shadowed by the X-ray chamber. To compensate for such effects, we initially tested separating the individual leaves before any image adjustment and then applying individually adjusted filters to each of the images. However, this approach was skipped because the gain in image quality was disproportionate to the amount of time spent on image adjustments (i.e. approximately a 10-fold increase in image processing time).

## Discussion

**Fig. 7:**
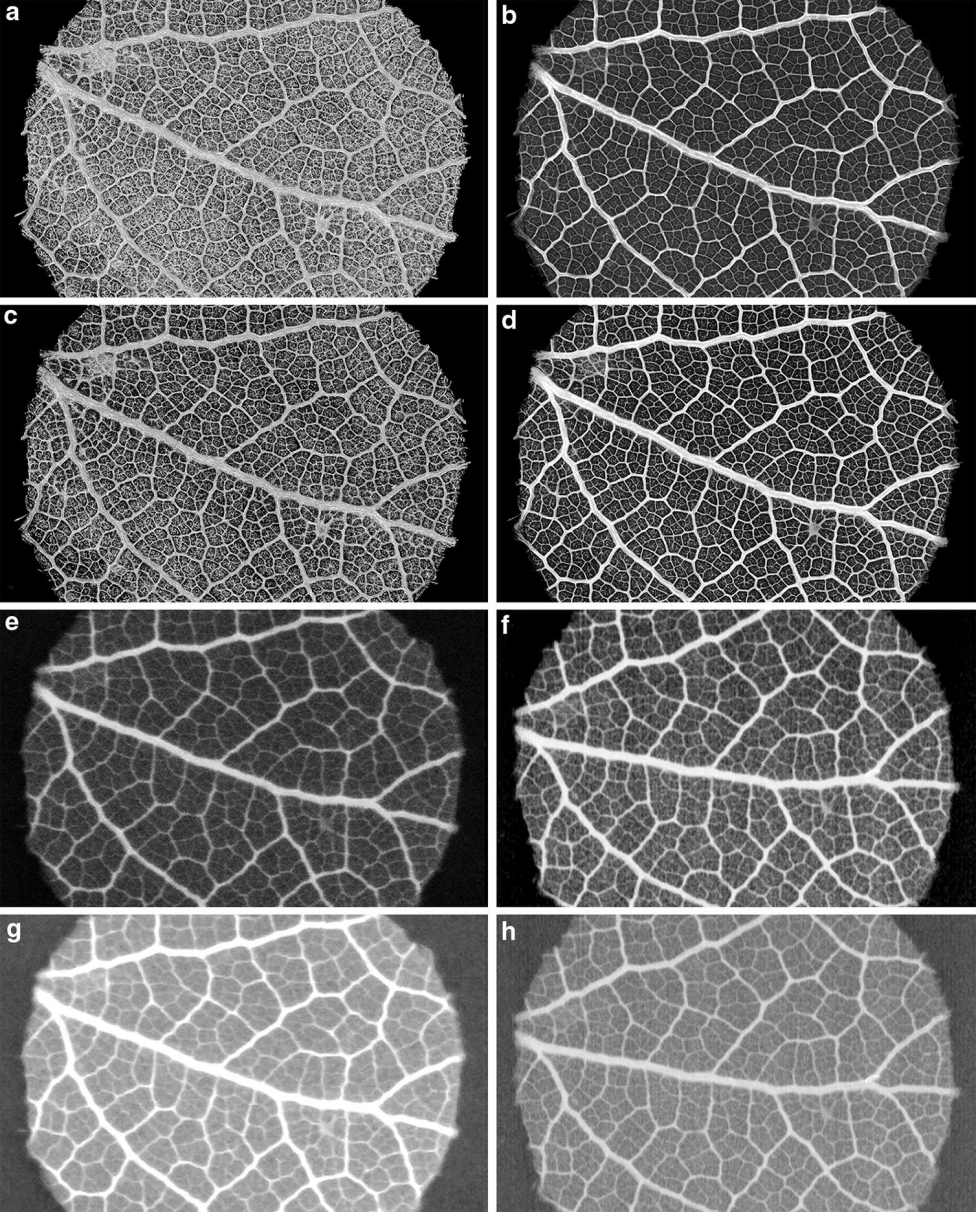
Comparison of 3D and virtual 2D µCT (**a**–**d**) with 2D contact microradiography images (**e**–**h**) of *Quercus faginea* (Fagaceae) and the influence of different image plate resolutions and processing standards, all shown as X-ray negatives. **a** Penultimate step of the 3D data processing, in which non-target voxels, the epidermis and approximately 50% of the mesophyll tissue were removed during segmentation (modus: “Volume renderer Phong” with slight Erode-settings). The leaf venation was only eroded in the outermost layer but generally difficult to differentiate from surrounding parenchyma. This is due to the volume rendering procedure, which always represents rendering of surfaces. Thus, thickness information by density signals is lost. **b** Penultimate step of the 3D µCT image processing as a virtual 2D X-ray image (2D mode: “Sum along Ray” or—if more appropriate—“Maximum Projection”). The density values added up along a line of vision through the selected sub-volume showing that the highest vein orders are easier to discern from the surrounding parenchyma than in the 3D model due to particular density differences. **c** Final step of the 3D µCT image as a surface model (viewed from abaxial leaf surface). The skeletonization of the leaf venation is maximized by increasing the erosion with a slightly higher threshold. Part of the parenchyma is still visible and cannot be further faded out without impairing vein connectivity. **d** Final step of the 3D µCT image in virtual 2D X-ray mode. This step consists in the individually adjusted increase in contrast of the lower density values (dark tones) at the cost of a loss in contrast in the higher density values (light tones). The non-veinous mesophyll exhibits low density values (except for some granular structures) that are not confluent with the veins. Therefore, a high contrast of the leaf venation up to the highest vein orders is achieved. This processing mode is used subsequently as the reference µCT image. **e** Individual adjustment of the 2D X-ray image (20 µm) with better contrast and lower artificial widening (dilatation) of the veins compared to the standard protocol. **f** Individual adjustment of the 2D X-ray image (7 µm resolution). The increased resolution of the vein network compared to the industrial standard image is evident. Linear artefacts are visible which can be easily discerned from the veins. **g** Industrial standard (25 µm resolution) 2D X-ray image with simple contrasting according to our standard protocol. **h** Image processing of the 7 µm project standard (2D X-ray) according to our standard protocol. Leaf fragment 1 cm wide

Here, micro CT with a resolution of 6.25 µm proved suitable for high resolution imaging of leaf venation. For most samples, these images provided sufficient resolution and contrast to determine the connectivity of veins, thus allowing for accurate measurements of vein density (e.g. Figs. 5, 6). Blonder et al. [6] also used micro CT but had difficulties to determine the connectivity of the veins. This might have been due to the small amount of samples tested (only two leaf samples were processed), but most likely was due to the lower resolution (15 µm) of their system which was limited by the effective size of the focal spot. Another reason could be the different method of image processing. In their study, local contrast was enhanced using a contrast-limited adaptive histogram equalization following Zuiderveld [26]. However, it is unclear how the image segments were processed to obtain a 2D view of the venation. We tested different image processing methods and achieved highest contrast of leaf venation using a virtual 2D processing of the CT raw data. Compared to the 3D images, virtual 2D had a much lower background of mostly parenchymatic mesophyll with our approach (for details see Fig. 7).

For practical reasons we selected a resolution of 6.25 µm for our micro CT system. This allowed us to use sample sizes that are large enough for vein density measurements and keep the file size and scan times at reasonable scales. However, our dataset showed that full resolution of the leaf venation architecture and connectivity was not achieved in all samples. One factor was the shading by non-veinous structures such as leaf crystals (Fig. 6), which is not only a problem for X-ray imaging. Lack of full vein connectivity in some samples arose due to weak contrast of the free-ending last order veins (FEV). Such a problem might be surmounted by slightly increasing the image resolution. However, changing the geometry of our X-ray system to achieve higher resolution, for example to sub-micron dimensions, most likely introduces more background noise due to increased resolution of cellular structures [14] and is not expected to be useful. Moreover, changing the geometry would also come at the cost of reduced sample size with the consequence that the imaged section of the vein network may become too small for representative replicate vein measurements. Therefore, we emphasize that our approach with a resolution of 6.25 µm will most likely be adequate for the majority of the leaf samples, especially of woody species with a higher degree of vein-forming lignified tissues.

Compared with chemical leaf clearing and subsequent imaging using light microscopy or a flatbed scanner (the latter for entire leaves), our micro CT imaging approach has the advantage of being not only non-destructive but also of providing images that are often better suited for automated image analysis because of rather uniform contrast, homogeneous sharpness and the absence of preparation artefacts as often observed with leaf clearing (e.g., inhomogeneous staining, disturbing non-veinous structures). The stronger the contrast and the higher the uniformity of vein “staining” across the sample, the easier the semi-automatic generation of vein skeletons as for example with LeafGUI ([5); for potential artefacts, see [24]) or phenoVein [27]. This is particularly important because advancing the exploration of vein data not only requires improvements in imaging methods but also in the degree of automation of image analysis and data extraction [28].

A drawback of very high resolution micro CT is still the long, usually multi-hour scan time per sample. Here, we significantly reduced the per sample scan time by processing multiple samples during a single scan. Given the chosen geometric setting and the focal spot size of our X-ray system, up to about 15 samples could be scanned with sufficient image quality and intersample distance that is required for sample separation during 3D rendering. A standard 3D rendering of leaf surfaces is of limited value for vein analyses compared to the production of 2D radiographies from the 3D image stack, because these 2D images are much better suited to show vein architecture and can be directly compared with the microscopic images. However, 3D rendering program algorithms are also required prior to obtaining 2D radiographies from CT-data (see methods) and thus cannot be skipped with the aim of saving processing time. Larger sample numbers led to samples at the (upper and lower) extremes of the field of view that were out of the optimal 3D-reconstruction area. However, if a helix CT system were available, a much higher number of samples could be processed with that method.

Our standard for 3D rendering further facilitates image processing in micro CT based visualization of vein networks. Nevertheless, the computation-intensive 3D rendering is the remaining bottleneck that prevents a higher throughput with micro CT, an obstacle that might be circumvented with massive parallel computing facilities and manpower.

A significant progress in non-destructive rapid imaging of leaf venation is our 2D X-ray method with a resolution of 7 µm, which is a 3.6-fold increase in effective resolution compared to the industrial standard. Although the resolution of the venation architecture is still lower than with micro CT, this 2D approach permits the processing of up to two hundred medium-sized leaf samples per week at an image quality that is sufficient for many research fields (e.g. systematics and evolution, paleobotany) and that is superior to the industrial standard resolution that has prevailed to date [6]. In spite of the strong increase in resolution to dimensions that should be sufficient for visualizing also the highest vein orders in most cases, the low contrast between vein and surrounding mesophyll tissue remains a challenge for imaging and analysis of the smallest veins.

Besides its suitability for higher throughput, another major advantage of 2D X-ray imaging is that entire leaves up to dimensions of 18 × 24 cm or 10 × 12 cm (that is the maximum available size of the image plates for the 20 µm or 7 µm approaches, respectively) can be imaged. Only entire leaves allow for length measurements of major veins (i.e. vein orders 1°–3°), which is required for the determination of total vein density, a trait often used in macroecology [1, 29]. Entire leaves can be also imaged with micro CT [6], but at the cost of image resolution and processing speed. For studies that require the determination of total VLA, we suggest to start with rapid non-destructive 2D X-ray imaging with 7 or 20 µm resolution and, if the resolution of the highest vein orders is insufficient, to add micro CT for amending minor vein density. If adequate micro CT systems are not available, minor VLA can still be determined alternatively from small chemically cleared leaf fragments. Small fragments are informative enough, more rapidly processed, and are more readily permitted to be extracted from herbarium specimens than entire leaves (while entire leaves used for non-destructive imaging of major veins can be re-inserted on the herbarium voucher).

## Conclusions

We advanced X-ray based visualization of leaf venation networks following two lines, one that produces very high resolution images using micro CT to generate a non-destructive alternative to traditional destructive vein imaging, and a second one that is suited for rapid imaging and routine mass screening of, for example, herbarium collections but at a higher image resolution than the industrial standard. This overall gain in resolution translates into an increased precision of measurements of vein lengths and diameters and a higher proportion of vein traits recovered from the images. Therefore, these non-destructive approaches will aid in vein trait data mining from museum collections, which provide an immense and invaluable treasure of still underused historical and recent information on environmental and evolutionary change. To further improve throughput, future studies will also have to advance the automation of vein data extraction and pattern recognition from images. Particularly promising are approaches based on computer vision and machine learning as recently shown [28].

## Additional files

**Additional file 1: Fig. S1.** Scheme of comparative vein density measurements in 25 µm and 7 µm images with identical reference areas (here based on *Bridelia ferruginea*).

**Additional file 2: Fig. S2.** 2D image from 3D-CT-volume data of *Bauhinia rufescens*. Note rarifications of leaf venation in the dark (empty) areas as a consequence of insufficient reconstruction.

## Abbreviations

2D/3D: 2-/3-dimensional
FEV: free-ending veins
Micro CT: microfocus X-ray computed tomography
Minor VLA: length of minor veins (i.e. vein orders 4° and higher) per unit area
VLA: vein length per unit area (i.e. vein density)
