# Supplementary figures and images for: Improved non-destructive 2D and 3D X-ray imaging of leaf venation
Source: Plant Methods. 2018 Jan 19;14:7. doi: 10.1186/s13007-018-0274-y (PMC5774031; doi:10.1186/s13007-018-0274-y)

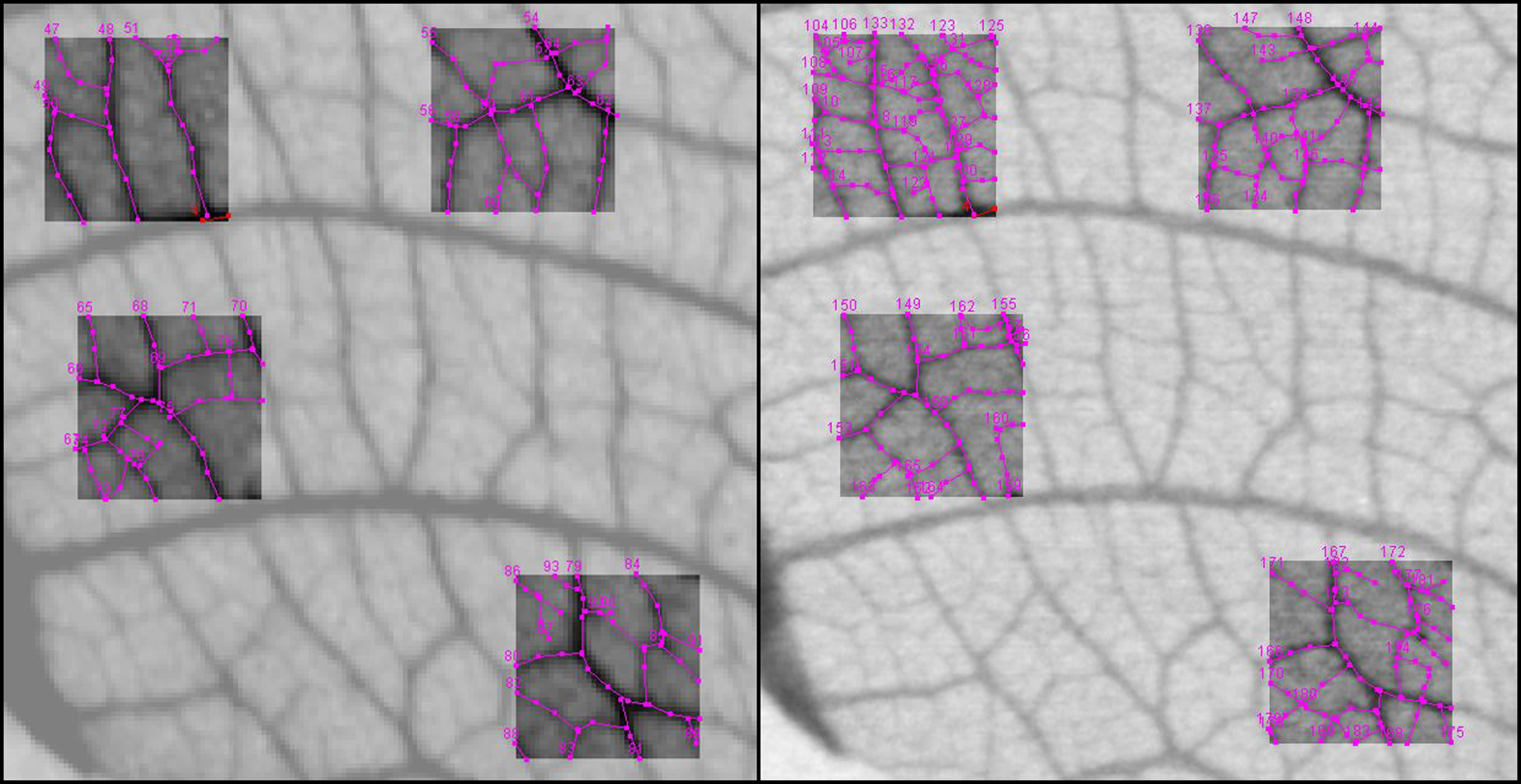

Supplement: Supplementary file 1 — Additional file 1: Fig. S1. Scheme of comparative vein density measurements in 25 µm and 7 µm images with identical reference areas (here based on Bridelia ferruginea). [file 13007_2018_274_MOESM1_ESM.jpg]

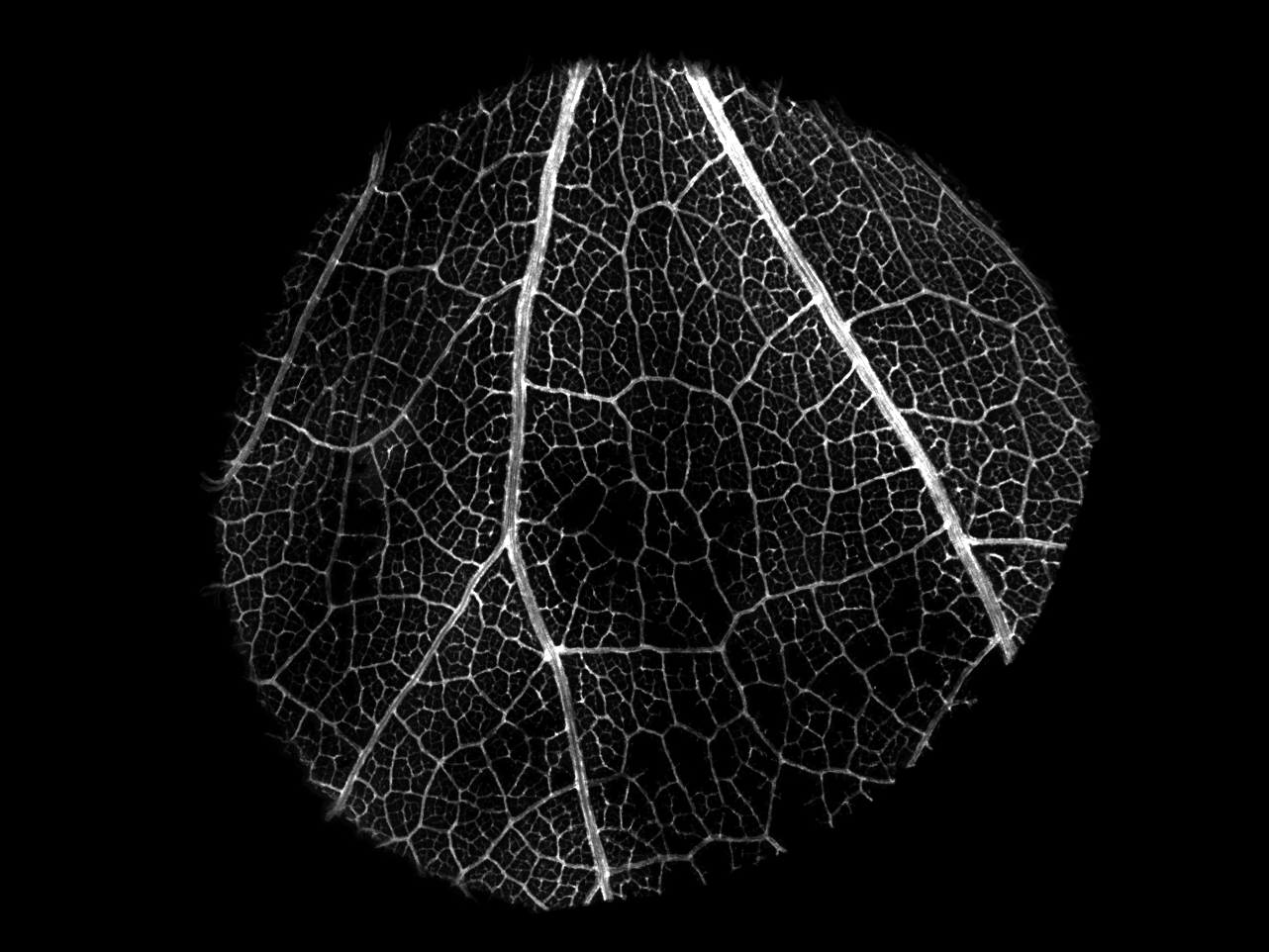

Supplement: Supplementary file 2 — Additional file 2: Fig. S2. 2D image from 3D-CT-volume data of Bauhinia rufescens. Note rarifications of leaf venation in the dark (empty) areas as a consequence of insufficient reconstruction. [file 13007_2018_274_MOESM2_ESM.tif]
